# Hemoglobin-Based Oxygen Carriers: Potential Applications in Solid Organ Preservation

**DOI:** 10.3389/fphar.2021.760215

**Published:** 2021-11-30

**Authors:** Min Cao, Guoqing Wang, Hongli He, Ruiming Yue, Yong Zhao, Lingai Pan, Weiwei Huang, Yang Guo, Tao Yin, Lina Ma, Dingding Zhang, Xiaobo Huang

**Affiliations:** ^1^ Department of Critical Care Medicine, Sichuan Provincial People’s Hospital, University of Electronic Science and Technology of China, Chengdu, China; ^2^ Anesthesiology, Southwest Medicine University, Luzhou, China; ^3^ Surgical Department, Chengdu Second People’s Hospital, Chengdu, China; ^4^ Health Inspection and Quarantine, Chengdu Medical College, Chengdu, China; ^5^ Sichuan Provincial Key Laboratory for Disease Gene Study, Sichuan Provincial People’s Hospital, University of Electronic Science and Technology of China, Chengdu, China

**Keywords:** hemoglobin-based oxygen carriers, solid organ preservation, machine perfusion, organ oxygenation, ischemia–reperfusion injury

## Abstract

Ameliorating graft injury induced by ischemia and hypoxia, expanding the donor pool, and improving graft quality and recipient prognosis are still goals pursued by the transplant community. The preservation of organs during this process from donor to recipient is critical to the prognosis of both the graft and the recipient. At present, static cold storage, which is most widely used in clinical practice, not only reduces cell metabolism and oxygen demand through low temperature but also prevents cell edema and resists apoptosis through the application of traditional preservation solutions, but these do not improve hypoxia and increase oxygenation of the donor organ. In recent years, improving the ischemia and hypoxia of grafts during preservation and repairing the quality of marginal donor organs have been of great concern. Hemoglobin-based oxygen carriers (HBOCs) are “made of” natural hemoglobins that were originally developed as blood substitutes but have been extended to a variety of hypoxic clinical situations due to their ability to release oxygen. Compared with traditional preservation protocols, the addition of HBOCs to traditional preservation protocols provides more oxygen to organs to meet their energy metabolic needs, prolong preservation time, reduce ischemia–reperfusion injury to grafts, improve graft quality, and even increase the number of transplantable donors. The focus of the present study was to review the potential applications of HBOCs in solid organ preservation and provide new approaches to understanding the mechanism of the promising strategies for organ preservation.

## Introduction

Solid organ transplantation (SOT) is an optimal, lifesaving treatment choice for patients with end-stage organ failure. The number of transplants has increased dramatically in the last decade, but there remains a considerable imbalance between the demand and supply of donor organs worldwide (OPTN/SRTR 2019 Annual Data Report, 2021a, 2021b). According to the data from the OPTN/SRTR 2019 Annual Data Report about Deceased Organ Donation, donations after circulatory death (DCD) increased to 32,313 in 2019 from 29,675 in 2018 (Israni et al., 2021). Ischemia–reperfusion injury (IRI) unavoidably occurs during organ resection and transplantation, may compromise the short-term and long-term after transplantation, and remains a critical organ transplantation challenge. With the increase in expanded criteria donor (ECD), the selection of better preservation methods to improve tissue oxygenation during isolated organ preservation to further reduce IRI of organs remains a pressing issue.

Ischemia and hypoxia are the leading causes of graft damage during the storage of isolated organs and may lead to a variety of destructive effects, such as ion imbalance, uncoupling of mitochondria, activation of endothelial cells, activation of various cell death programs, and proinflammatory immune responses (Kalisvaart et al., 2018; Lavallard et al., 2020). The current static cold storage (SCS) of grafts reduces enzyme activity and oxygen consumption, but the demand for energy metabolism and oxygen continues while cells maintain ion homeostasis and membrane potential (Kasil et al., 2019; Ali et al., 2020). Several methods for supplementing oxygen in organ preservation solutions have been reported. One method is the direct infusion of gaseous oxygen through mechanical perfusion platforms using various techniques, such as retrograde perfusion and supplemental oxygen under high pressure conditions (Higuchi et al., 2006; Minor et al., 2011; Tran et al., 2012). Supplemental oxygen during organ preservation promotes mitochondrial adenosine triphosphate (ATP) synthesis, prevents anaerobic metabolism, and reduces reperfusion injury (Minor et al., 2011). However, if the oxidative metabolism of the graft is severely impaired, direct oxygenation may exacerbate the oxidative stress damage to the organ. Another approach is to use oxygen carriers such as red blood cells (RBCs), humans, animals, recombinant sources, and perfluorochemicals (PFCs) (Tsujimura et al., 2002; Sakai et al., 2011; Teh et al., 2017; Liu et al., 2021). Perfusion of organs with blood as an oxygen carrier has been shown to reduce IRI to organs and improve grafts’ quality and short-term prognosis. However, blood has some potential disadvantages, including immune phenomena, blood infection, cross-matching and scarcity, and it can only be used for subnormothermic machine perfusion (SNMP) and normothermic machine perfusion (NMP) because RBCs can undergo hemolysis in a low-temperature environment (Cai et al., 2020; Lv et al., 2021). PFCs are hydrophobic, lipophilic, and difficult to disinfect, making it difficult to promote their use in clinical applications (Partlow et al., 2008). Therefore, researchers have been trying to find solutions that can provide oxygen close to physiological conditions during the preservation of isolated organs. Hemoglobin-based oxygen carriers (HBOCs) are a class of oxygen carriers that bind and release oxygen closer to physiological conditions than that of perfluorocarbons.

HBOCs are cell-free carriers obtained by processing free hemoglobin via chemical modification, genetic modification, or embedding (Standl et al., 2003). The oxygen affinity of some HBOCs is lower than that of human hemoglobin, thus facilitating oxygen release to tissues as the hemoglobin dissociation curve shifts to the right (Page et al., 1998). HBOCs were initially investigated as a blood substitute and have now been expanded for ischemic and hypoxic treatments. The application of HBOCs to the preservation of isolated organs may have several potential advantages: maintenance of cellular metabolic needs and mitigation of hypoxic damage; much lower diameter than that of RBCs for better oxygen delivery; no immune reactivity; the needs of the period of organ preservation; good stability for long storage time; wide range of temperature adaptation; and easy availability from a wide variety of sources. The effectiveness and safety of HBOCs in the preservation of grafts have been studied in numerous preclinical and clinical studies. This review summarizes the characteristics of HBOCs and presents recent advances in the preservation of solid organs in preclinical and clinical studies with the addition of HBOCs to the preservation solution.

## Overview of HBOCs

HBOCs are biological products obtained from purified hemoglobin (Hb) to have the ability to bind and release oxygen. Hb is a tetrameric protein molecule of ∼64 kDa consisting of two noncovalently bound αβ dimers (Sahu et al., 2006). Free Hb released from aged or damaged RBCs dissociates in dimers that in plasma have a half-life only of a few hours (Bleeker et al., 1989). The released heme causes lipid peroxide- or H_2_O_2_-driven formation of ferryl myoglobin (Mb), which generates radicals and contributes to renal dysfunction (Moore et al., 1998; Alayash et al., 2007). Moreover, dimers are ultrafiltered in the kidneys, causing nephrotoxicity (Simoni et al., 1997). Hb cross-linking, polymerization, and conjugation to polymers prolong intravascular half-life, stability, and safety (Adachi et al., 1987; Bobofchak et al., 2003; Gordon et al., 2005). HBOCs were initially developed as blood substitutes. Numerous preclinical and clinical studies initially explored the safety and efficacy of HBOCs. HBOC-201 has been available in South Africa since 2001 and is approved for surgical patients with acute anemia (Mer et al., 2016). However, in 2008, Dr. Natanson and others included 13 randomized controlled trials in a meta-analysis of cell-free hemoglobin-based blood substitutes and concluded that the use of HBOCs was associated with a significantly increased risk of death and myocardial infarction (MI) based on an analysis of the available data (Natanson et al., 2008). Subsequently, the Food and Drug Administration (FDA) suspended all HBOC trials in the United States. The study of this meta-analysis generated widespread controversy. Related editorials suggested that if the imminent risk of death due to low Hb outweighed the risk of HBOCs, requiring a moratorium on all HBOC trials could be fatal for these patients (Levien et al., 2008; Sarani and Gracias, 2008; Sauaia et al., 2008). Since 2014, clinical trials for HBOCs are currently available only through expanded access (EA) to save patients’ lives when other interventions are not available (Mackenzie et al., 2010). HBOCs are presently approved for veterinary use in the United States, Russia, and the European Union (Jahr et al., 2008). Owing to the ability to bind oxygen and release oxygen, HBOCs have been extended for applications such as protection of isolated organs (van Leeuwen et al., 2019), initiation of extracorporeal membrane oxygenation (ECMO) circuit as the pump-priming solution (York et al., 2002; Henderson et al., 2004), tumors (Belcher et al., 2020), and other ischemic and hypoxic diseases.

Based on the different sources of Hb, the main HBOCs applied for organ preservation are marine lugworm *Arenicola marina* (M101) (*Arenicola marina*), HBOC-201 (bovine), hemoglobin-vesicles (HbV) (human), polymerized human placenta hemoglobin (PolyPHb) (human), and hemoglobin glutamer (HbG) 200 (bovine). An overview of the different oxygen carriers used in solid organ preservation is provided to understand their properties better (Table 1). HBOCs of different origins have commonalities and dissimilarities. HBOCs have the advantages of no cell membrane, no blood type, no cross-matching requirements, no risk of viral infection, long-term storage, and timely availability, so they are excellent options for the preservation of isolated organs (Caswell et al., 2005). The molecular diameters of several HBOCs used for organ preservation (8–250 nm) are much smaller than those of RBCs (7,000 nm) and their viscosity is lower, thus making it easier for HBOCs not only to enter tissues through blood vessels and improve tissue oxygenation but also to promote oxygen diffusion in the microcirculation, resulting in a uniform distribution in the graft (Scatena and Giardina, 2001). Due to the different sources of Hb, different HBOCs also have their unique characteristics. M101 is a natural extracellular biopolymer Hb obtained from a marine invertebrate, *Arenicola marina*. M101 has a 3,600-kDa structure composed of 156 globins and 44 nonglobin linker chains that can carry up to 156 O_2_ molecules when saturated (Toulmond et al., 1990; Zal et al., 1997; Rousselot et al., 2006). M101 possesses intrinsic Cu/Zn-superoxide dismutase antioxidant activity, protecting tissue from superoxide radicals to a certain extent (Rousselot, et al., 2006). M101 is formulated as a good manufacturing practice (GMP)–compliant commercial class III medical device that is used as an additive to existing organ preservation solutions. HBOC-201 [hemoglobin glutamer-250 (bovine); Hemopure, HbO_2_ Therapeutics LLC, Souderton, PA 18964, United States] is derived from bovine RBCs. Free Hb is then purified by chromatography and cross-linked with glutaraldehyde (GDA) to increase the stability and molecular size (Hughes et al., 1995; Callas et al., 1997). When fully saturated, HBOC-201 binds approximately 1.36 ml oxygen per gram of Hb. Since the Hb dissociation curve is shifted to the right, it releases oxygen more easily than human Hb (Callas et al., 1997; Dubé et al., 2008). PolyPHb is purified from fresh human placental blood, heat-treated to inactivate it, and then intramolecularly and intermolecularly cross-linked with pyridoxal phosphate (PLP) and GDA. Then, ultrafiltration and molecular sieve chromatography are performed to obtain polyhemoglobin with a molecular weight of 64–600 kDa (Li et al., 2009). The HbV is a cellular-type HBOC involving polyethylene glycol (PEG), in which phospholipid vesicles encapsulating nearly 35–40 g/dl human Hb are embedded; the Hb concentration of the HbV suspension is 10 g/dl. One HbV particle (∼250 nm diameter) contains approximately 30,000 Hb molecules (Sakai et al., 1996; Sakai et al., 2000; Sakai et al., 2001).

**TABLE 1:** Overview of different oxygen carriers in solid organ preservation.

Product	Current status	Company	Source of Hb	Modification	Half-life	Size	Average particle diameter	P_50_	Shelf life
M101	Clinical	Hemarina, Morlaix, Brittany, France	Marine Isnvertebrate (Arenicola marina)	Hexagonal bilayer-linked globin molecules	2.5 days	3,600 kDa	25 nm	7 mmHg	N
HBOC-201	Clinical	Acquired by Hemoglobin Oxygen Therapeutics in 2014, Souderton, PA	Bovine	Glutaraldehyde polymerization	19–24 h	250 kDa	8 nm	40 mmHg	3 years
HbV	Preclinical	Waseda Univ. & Keio Univ; Nara Medical University (2013)	Human	Polyethylene glycol (PEG) chains	15–20	N	250 nm	9 mmHg	2 years
polypHb	Experimental	Tianjin Union Stem Cell Genetic Engineering Ltd., Tianjin, China	Human	Cross-linkage with glutaraldehyde	N	64–600 kDa	N	5–9 mmHg	N
HbG-200	Preclinical	Oxyglobin^®^, Biopure, Cambridge, MA	Bovine	Glutaraldehyde-polymerized	18–43 h	200 kDa	N	34 mmHg	N
Human red blood cells	Clinical	N	Human	N	120 days	64 KDa	7000 nm	27 mmHg	3 weeks

N, not reported; HbV, hemoglobin-vesicles; PolyPHb, polymerized human placenta hemoglobin; HbG, hemoglobin glutamer.

According to previous studies, direct infusion of HBOCs in the body can cause vasoconstriction, methemoglobin, and other side effects. The causes of vasoconstriction may include the following factors: scavenge nitric oxide (NO) (Standl, 2005), upregulated endothelin (ET) production (Taverne et al., 2017), the low viscosity and shear stress (Pearce and Gawryl, 2003), and vessel wall hyperoxygenation (Standl, 2005; Cabrales et al., 2009), of which the scavenging of NO is the leading cause of vasoconstriction. Hb in RBCs not only binds and transports oxygen in the circulation but also is an efficient scavenger of NO (Doyle and Hoekstra, 1981). However, the NO-scavenging rate of Hb in RBCs is reduced by a red cell–free zone along the endothelium in the blood, extracellular diffusion of NO to RBCs, and reduced NO diffusion over RBC membrane. All three of these mechanisms are eliminated by direct intravascular infusion of HBOCs, and the high affinity and low shear stress of HBOCs for NO also lead to a reduction in circulating NO (Jeffers et al., 2006). Thus, in the presence of free Hb, bioavailable NO is reduced and leads to vasoconstriction and hypertension, increased proinflammatory mediators and vasoconstrictive factors, and platelet inactivation (Loscalzo, 1997; Rother et al., 2005).

Although there are differences in these vascular parameters, there are few reports on vasoconstriction damage in the preservation of isolated organs. The following reasons may underlie the relative lack of reports: carbon monoxide (CO) production is increased during the degradation of heme (Alayash, 2017; Zhu et al., 2019); HBOCs provide a sufficient oxygen supply to cells, which may balance the effects of vascular parameters (Gould et al., 1998); the concentration of HBOCs during the preservation of isolated organs is much lower (de Vries et al., 2019); compared to protecting isolated organs from ischemia–reperfusion injury, the advantages of HBOCs outweigh the disadvantages (Topp et al., 2008); the lavage fluid neutralizes the HBOCs before they reach the recipient; and the concentration of HBOCs that eventually reaches the circulation is negligible (Mahboub et al., 2020). Unlike RBCs, HBOCs do not contain NADH-dependent methemoglobin reductase, which is responsible for converting methemoglobin back to Hb. The reversible oxygen binding of Hb to oxygen depends on the reduced state of iron atoms in the structure of Hb. However, one or more iron ions in methemoglobin are in the oxidized state and are unable to bind oxygen while increasing the affinity of Hb for oxygen and shifting the oxygen dissociation curve to the left, resulting in decreasing its ability to release and deliver oxygen (Dixit et al., 2021). However, elevated methemoglobin in the recipient has rarely been reported when HBOCs are applied to preserve grafts. This is possible because methemoglobin is washed out of the perfusion fluid before transplantation, making the amount of methemoglobin that eventually reaches the recipient’s circulation negligible. Moreover, supplementation with additional HBOC-201, glutathione, vitamin C, and methylene blue also corrected or slowed the increased percentage of methemoglobin caused by HBOCs (de Vries et al., 2019; Mackenzie et al., 2010; Unnikrishnan et al., 2019). Therefore, applying conventional preservation solutions with added HBOCs to the preservation of isolated organs can adequately improve the oxygenation of tissues with fewer side effects. More importantly, more randomized clinical trials with large samples are needed to validate the safety and efficacy of HBOCs in SOT.

## HBOCs in Solid Organ Transplantation

**FIGURE 1:**
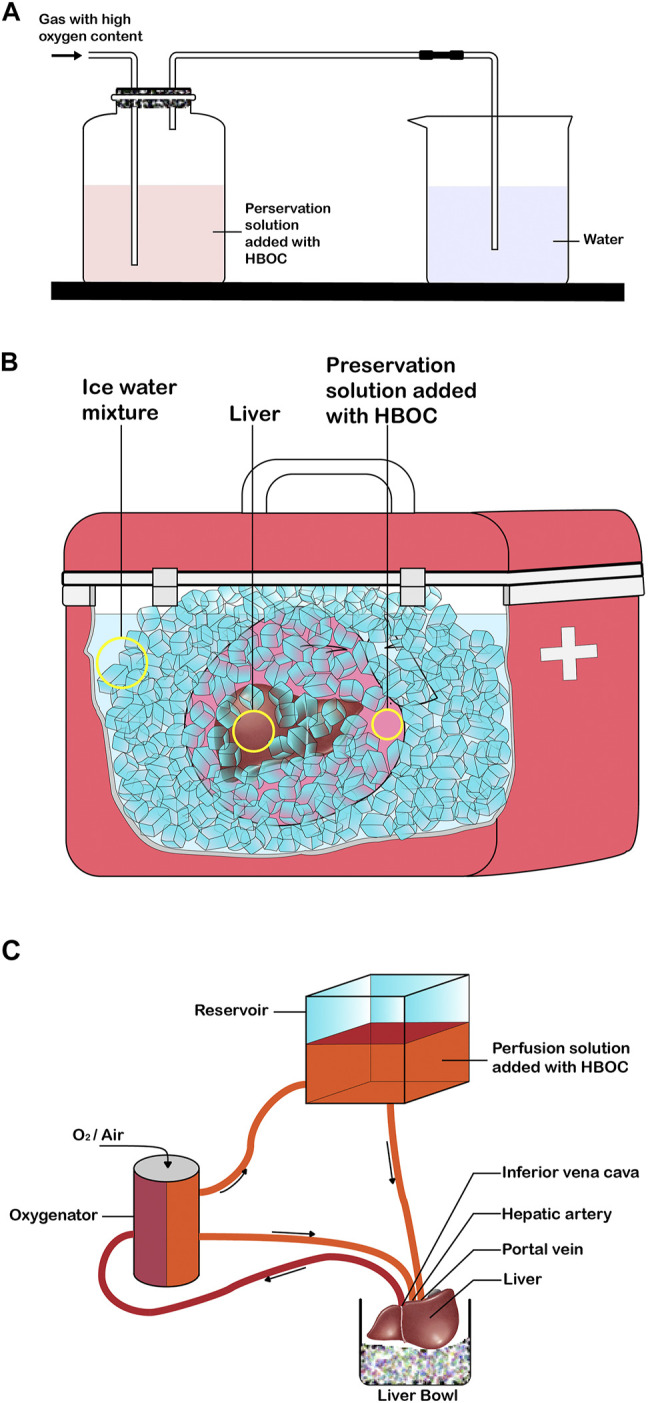
Application of HBOC in solid organ preservation (e.g., liver). **(A)** Pre-oxygenation process of preservation solution added with HBOC. **(B)** Static cold preservation of isolated liver after pre-oxygenation with the preservative solution of added HBOC. **(C)** Mechanical perfusion of the isolated liver after pre-oxygenation with the preservation fluid of added HBOC.

**FIGURE 2:**
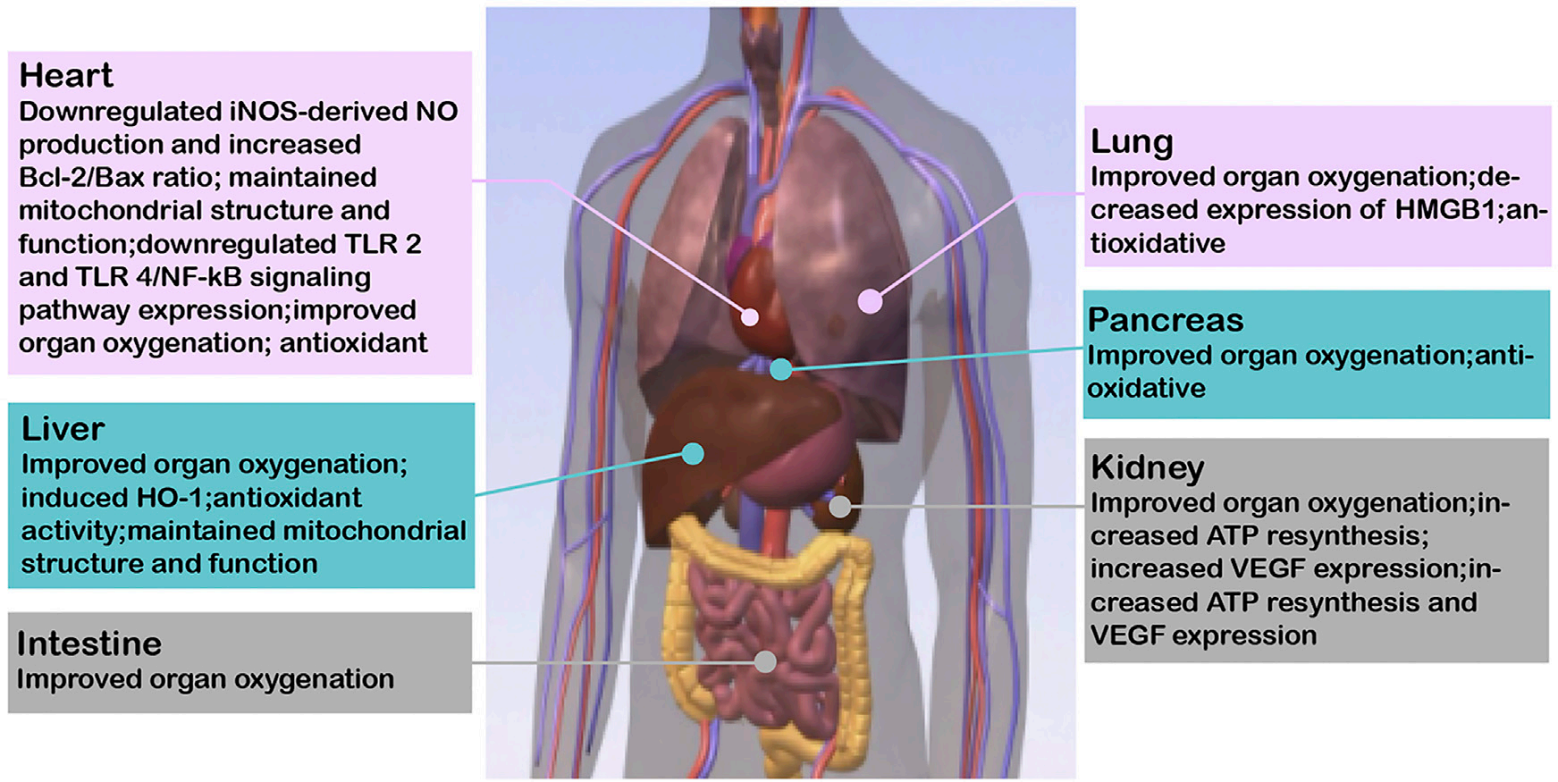
Relevant mechanisms where HBOCs are beneficial to the preservation of solid organs. iNOS, inducible nitric oxide synthase; NO, nitric oxide; Bcl-2, B-cell lymphoma-2; TLR, toll-like receptor; NF-kB, nuclear factor-k-gene binding; HO-1, heme oxygenase-1; ATP, adenosine triphosphate; VEGF, vascular endothelial growth factor.

The preservation of isolated organs is necessary before transplantation into a recipient’s body. Thus, the choice of preservation solution is crucial for the protection of isolated organs. Countless traditional preservation solutions were developed with the increase in SOT in the 1970s, such as Euro-Collins (EC) (Ingemansson et al., 1995), Celsior, University of Wisconsin (UW), and histidine-tryptophan-ketoglutarate (HTK) solutions (Warnecke et al., 2002; Yoshida et al., 2002). The current mainstream organ preservation protocol is still to preserve organs in a standard conventional solution at 4°C. The purpose of preserving grafts in standard preservation solutions is to slow down the metabolic rate of tissue cells, maintain the balance of substances inside and outside the cells, and reduce cell swelling (Gao et al., 2015; Tian et al., 2009). To further improve graft quality and recipient prognosis, several studies have reported the addition of sugars (Kobayashi et al., 1991), calcium ion antagonists (Ingemansson et al., 1996), potassium channel openers (Han et al., 2009), and platelet-activating factor antagonists (Kim et al., 2000) to the standard preservation solutions. However, the addition of these substances did not improve oxygenation of the tissues. The most fundamental cause of graft injury after removal from the donor is caused by ischemia and hypoxia. The conventional preservation solution with the addition of HBOCs is preoxygenated for a period of time, and the oxygenated solution will be used for static cold preservation and/or mechanical perfusion of the organ (Figure 1A). HBOCs exhibit good compatibility with traditional preservation solutions, and there is no need to modify the characteristics and protocols of organ preservation. Conventional preservation solution with HBOC added performs static cold preservation of the donor organ during the cold ischemic time (time from the perfusion of the organ with the solution to its removal from the cold storage for implantation) (Nakamura et al., 2020), a process that not only reduces cellular energy metabolism but also provides oxygen to meet organ oxygen demand (Figure 1B). The preservation solution containing HBOCs reduces ischemic and hypoxic damage to isolated organs, prolongs the preservation time of organs to meet long-distance transfer, and improves the utilization of organs to some extent. With the advancement of preservation technology and machines that simulate clinical reperfusion, isolated organs can be repaired and evaluated using the MP (machine perfusion) platform after long-distance cold preservation and transportation, which is especially beneficial for the use of marginal organs (Grundmann et al., 1977). Conventional preservation solutions with the addition of HBOCs are combined with different types of MP (hypothermic machine perfusion (HMP), SNMP, NMP, DHOPE [(dual) hypothermic oxygenated machine perfusion–controlled oxygenation rewarming (COR)-NMP]) for the repair and evaluation of marginal organs, which can improve the quality of marginal organs and patient prognosis, expanding to some extent the donor pool (Figure 1C). MP may be used as a platform for the dynamic circulation of isolated organs during preservation, and it induces a more even distribution of HBOCs in blood vessels, promotes the transport and diffusion of oxygen in the microcirculation, and improves the quality of the graft and the prognosis of the recipient (Fontes et al., 2015). Thus, the application of preservation solutions with HBOCs is similar to that of conventional preservation solutions and can be used directly for static cold preservation and mechanical perfusion of isolated organs without significant changes in protocol or equipment (Ali et al., 2020). HBOCs, as a class of oxygen carriers close to physiological conditions, can be used in various types of solid organs with multiple protocols for preservation. HBOCs are safe and feasible oxygen carriers that may be used for the preservation of organs *in vitro* (Figure 2). The application of HBOCs in different solid organs is discussed in detail below (Table 2).

**TABLE 2:** Studies evaluating the impact of HBOCs in solid organ transplantation.

Author and year	Oxygen carrier	Evaluated solution	Concen-tration	Oxyge-nation	Experimental proposal	Species	Results	Author conclusion	Limitation/toxicity
Heart									
Li et al. (2009) FREE RADICAL BIO MED 2009	PolyPHb	STS	5 g/L	Equilibrated with 95% O_2_ and 5% CO_2_ at 37°C for 30 min	Baseline (30 min; KHB); HS (8 h; 5 g Hb/dL PolyPHb + STS or blood or STS); 2 h KHB NMP	Sprague–Dawley rats	PolyPHb improved heart contraction, decreased infarction size and myocardial apoptosis, and maintained redox homeostasis	PolyPHb downregulated iNOS-derived NO production and increased Bcl-2/Bax ratio; preserved mitochondrial function; attenuated NO-mediated myocardial apoptosis; and restored the nitroso-redox balance	N
Li et al. (2010) FREE RADICAL BIO MED 2010	PolyPHb	STS	0.1 g/dl	Equilibrated with 95% O_2_ and 5% CO_2_ at 37°C for 30 min	Sham (150 min perfusion; KHB) WI (30 min baseline KHB; 35 min STS WI; 2 h KHB NMP); WI + HBOC (30 min baseline KHB; 35 min STS + HBOC WI; 2 h KHB reperfusion); HS (30 min baseline KHB; 8 h STS; 2 h KHB NMP); HS + HBOC (30 min baseline KHB; 8 h STS + HBOC; 2 h KHB NMP)	Sprague–Dawley rats	PolyPHb improved cardiac function; decreased myocardial infarction, necrosis, and apoptosis; elevated mitochondrial function; and did not change the mitochondrial structure	Attenuated mitochondrial oxidative damage; preserved mitochondrial E_h_ and SOD activity; preserved mitochondrial function; inhibited ROS burst; and depressed cytochrome c translocation	N
Wei et al. (2011) Cardiology 2011	PolyPHb	HTKs	0.3 g/dl	Equilibrated with 100% O_2_ at 37°C for 5 min	Sham (150 min KHB perfusion) HTK9 (30 min baseline KHB; 9 h HTKs HS; 2 h KHB NMP); HBOC9 (30 min baseline KHB; 9 h HTKs + HBOC HS; 2 h KHB NMP); HTK14 (30 min baseline KHB; 14 h HTKs HS; 2 h KHB NMP); HBOC14 (30 min baseline KHB; 14 h HTKs + HBOC HS; 2 h KHB NMP)	Sprague–Dawley rats	HBOCs improved heart contraction and decreased infarct size, necrosis, and apoptosis; reduced expression of Toll-like receptor 2 (TLR 2), TLR 4, TNF- a, IL-1β, and nuclear factor-kB activation	Downregulated TLR 2 and TLR 4/NF-kB signaling pathway expression	Inhibitors of TLR 2 and TLR 4 were not used; did not research the relationship between different doses of PolyPHb and TLR 2 and TLR 4/NF-kB signaling pathway expression
Teh et al. (2017) ARTIF CELL NANOMED B 2017	M101	Celsior	1 g/L	Equilibrated with 95% O_2_ and 5% CO_2_	4–8°C cold storage 8 h (Celsior; Celsior + M101); KHB 60 min NMP	Wistar rats	Coronary flow was significantly higher in M101 group; improved postischemic recovery of heart function	Improved organ oxygenation	N
You et al. (2011) ARTIF CELL BLOOD SUB 2011	PolyPHb	KHB	0.1 g/dl	Equilibrated with 95% O_2_ and 5% CO_2_ for 10 min	Sham (perfused by KHB 2 h) Control (40 min baseline; 45 min WI; 2 h NMP)	Sprague–Dawley rats	Deoxygenated HBOC pretreatment and ischemic preconditioning both equally improved the recovery of cardiac function and reduced the cardiac enzyme release and myocardial histopathology	Improved organ oxygenation	Ischemic preconditioning cannot block the aorta to induce cardiac ischemia; results cannot explain the cardioprotective mechanism
					Ischemic preconditioning (10 min baseline; three 5-min ischemia and 5-min KHB perfusion; 45 min WI; 2 h NMP) deoxy-HBOCs (10 min baseline; three 5-min deoxygenated HBOC perfusion and 5-min KHB perfusion; 45 min W; 2 h NMP)				
White et al. (2015) J HEART LUNG TRANSPL 2015	HBOC-201	STEEN Solution	40 g/L	N	RBC group (RBC + normal saline NMP 6 h); RBC + plasma group (whole blood NMP 6 h); HBOC group (HBOC-201 NMP 6 h); HBOC + plasma group (HBOC-201 + plasma NMP 6 h)	Domestic pigs	Whole blood–based perfusate minimized injury and provided superior preservation of myocardial function during NMP	HBOCs promoted spontaneous oxidation and ROS; HBOCs increased the proportion of methemoglobin and CVR; HBOCs increased troponin I levels and histologic myocardial injury scores	Did not directly quantify methemoglobin; did not directly test and confirm the efficacy of the filter in reducing circulating leukocytes. Methemoglobin mediated oxidative damage of endothelial cells
Lung									
Glorion et al. (2018) ARTIF CELL NANOMED B 2017	M101	PerfadexV R (Xvivo, Sweden)	2 g/L	N	Sham (similar to the recipient animals but without undergoing lung transplantation); Perfadex (4–6°C 24 h cold storage); M101 + Perfadex (4–6°C 24 h cold storage)	White pigs	M101 reduced graft vascular resistance and increased the graft oxygenation ratio	Improved organ oxygenation and decreased expression of HMGB1	N
Ali et al. (2020) J HEART LUNG TRANSPL 2020	M101 static cold storage (SCS) *ex vivo* lung perfusion (EVLP)	Low-potassium dextran solution (LPD)	1 g/L	N	LPD (SCS 36 h + EVLP 12 h); M101 + LPD (SCS 36 h + EVLP 12 h)	Yorkshire pigs	M101 provided better physiologic parameters and oxygenation; reduced edema formation and apoptotic cell death; improved tight junction preservation; reduced the level of IL-6 in recipient plasma	Improved organ oxygenation; antioxidative properties	Did not cause donor-related injuries before cold preservation; EVLP minimized the physiologic differences between the experimental groups; the optimal dose of the EOC and maximal tolerable ischemic times were not investigated
Liver									
Topp et al. (2008) J SURG RES 2008	HbG200	KH-Sol	3.3 g/dl	Equilibrated with 95% O_2_ and 5% CO_2_	KH-Sol (EC cold storage 24 h; rat blood + KH-Sol IPRL 180 min); KH-Sol + HbG (EC cold storage 24 h; rat blood + KH-Sol + HbG IPRL 180 min); KH-Sol.+6% HES (EC cold storage 24 h; rat blood and + KH-Sol + 6% HES IPRL 180 min); KH-Sol + HbG + SnPP pretreated (EC cold storage 24 h; rat blood and + KH-Sol + HbG + SnPP IPRL 180 min)	Wistar rat	HBOCs increased HO-1 expression and activity during reperfusion, which could be abolished by tin protoporphyrin IX application	Improved organ oxygenation and induced HO-1; the antioxidant and anti-inflammatory properties of HO-1	Endothelin-1–mediated vasoconstriction
Fontes et al. (2015) AM J TRANSPLANT 2015	HBOC-201	UW	3.5 g/dl	Oxygenated to SaO_2_ > 95% and paO_2_ > 400 mmHg	CSP (cold storage 9 h) MP (21°C, UW + HBOC 9 h)	Landrace pigs	MP/HBOC system had higher survival and superior graft function; oxygen delivered by the liver was 8 times that of oxygen intake; significantly improved liver preservation	Improved organ oxygenation	N
Matton et al. (2018) LIVER TRANSPLANT 2018	HBOC-201	20% Human albumin or 4% gelofusine	18.3 g/L	Equilibrated with 95% O_2_ and 5% CO_2_	RBC + FFP (6 h NMP); HBOC-201 + FFP (6 h NMP); HBOC-201 + gelofusine (6 h NMP)	Human	HBOC-201 had significantly higher hepatic adenosine triphosphate content, cumulative bile production, and portal and arterial flows	Improved organ oxygenation	Relatively small sample sizes; lack of transplant validation; study grouping was performed consecutively rather than after randomization
Laing et al. (2017) Transplantation 2017	HBOC-201	Modified Ringer’s lactate solution	63.7 g/L	Oxygenated to arterial pO_2_ = 20 kPa	HBOCs (Hemopure-based perfusion fluid 6 h NMP); RBCs (packed red blood cell–based fluid 6 h NMP)	Human	HBOC-perfused livers extracted more oxygen than those perfused with RBCs	Improved organ oxygenation; exhibited antioxidant activity	Livers were not transplanted. Did not simulate the reperfusion with whole blood containing immune cell populations
van Leeuwen et al. (2019). Ann Surg 2019	HBOC-201	UW	N	DHOPE: oxygenated to PaO_2_ > 80 kPa	1 h DHOPE; 1 h COR; 150 min NMP	Human	69% of livers that met all viability criteria were successfully transplanted; 100% graft survival at 3 and 6 months; DHOPE-COR-NMP increased the number of deceased donor liver transplants by 20%	Improved organ oxygenation	Lack of randomization; livers were not transplanted based on a low bile pH during NMP, and therefore have no follow-up
Shonaka et al. (2019) PLoS One 2019	HbV	UW	N	N	CS (WIT 1 h,CS 4 h); HMP (WIT 1 h, HMP 4 h); SNMP (WIT 1 h, SNMP 4 h); SNMP + HbV (WIT 1 h, SNMP + HbV 4 h)	Female cross-bred Large-Yorkshire, Landrace, and Duroc pigs	SNMP + HbV solution can reduce the reperfusion injury of DCD donor liver. The mitochondria, pH and lactate levels of the SNMP + HbV group were well maintained in comparison to the CS, HMP, and SNMP groups	Increased oxygenation and oxygen consumption, maintained mitochondrial structure and function, and had a protective effect on metabolic acidosis	Transplantation was not performed, and these results are not sufficient for predicting the effects of SNMP/HbV in a living body
Alix et al. (2020) JHEP Rep 2020	M101	UW	1 g/L	N	SCS (cold storage, UW 6 h); SCS + M101 (cold storage, UW+1 g/L M101 6 h); HOPE (HOPE, UW+1 g/L M101)	White/Landrace x Piétrain pigs	M101 effectively oxygenated liver grafts during preservation; prevented posttransplant injury without reaching the level of HOPE	Improved organ oxygenation and preserved ATP synthase activity	Two different solutions were used in the SCS and HOPE groups; no medicoeconomic data available regarding the potential benefit of M101 in liver transplantation
Boteon et al. (2019) PLoS One 2019	HBOC-201	UW	N	Equilibrated with 30% O_2_ 1–2 L/min	HOPE + NMP (UW + HOPE 2 h, UW + HBOC-201 + NMP 4 h); cold-to-warm group (UW + HBOC-201 + HOPE, gradually rewarmed, NMP for viability assessment, 6 h)	Human	Both HMP + NMP and cold-to-warm mitigated the oxidative-mediated tissue injury and enhanced hepatic energy stores; HMP + NMP simplified the logistics of this combination and was conducive to clinical applicability	Improved organ oxygenation and enhanced ATP synthesis	Relatively small sample sizes; organs were not transplanted; replacement of the perfusate may reduce toxic metabolites; unable to evaluate transaminases or other markers of oxidative stress in the perfusate; absolute values and proportional ATP increase were lower than those in other studies performing HOPE; lack of further data on the histology of the bile duct
de Vries et al. (2019) Am J Transplant. 2019	HBOC-201	UW	N	3-Month graft survival was 100%	DHOPE, 10°C 1 h; COR, the temperature was gradually increased approximately 1°C per 2 min to 37°C; NMP, 37°C 1 h	Human	The 3-month graft survival rate was 100%	Improved organ oxygenation	Relatively small sample sizes and lack of a control group; did not draw conclusions on the value of the COR phase; did not discriminate between the beneficial effects of DHOPE, COR, and NMP separately; HBOCs were converted to methemoglobin, especially in the venous phase
Shonaka et al. (2018) Transplant Proc 2018	HbV	UW	0.6–0.7 mg/dl	N	Group 1 (cold storage UW 240 min, reperfusion with autologous diluted blood 120 min); Group 2 (SNMP (20–22°C) UW 240 min, reperfusion with autologous diluted blood 120 min); Group 3 (SNMP HbV and UW 240 min, reperfusion with autologous diluted blood 120 min)	Female cross-bred Large-Yorkshire, Landrace, and Duroc pigs	HbV increased the oxygen consumption of the donor liver during SNMP	Improved organ oxygenation	Animal model; experiment on the transplant model, and therefore, the result of the actual transplant is unknown
Kidney									
Thuillier et al. (2011), AM J TRANSPLANT 2011	M101	UW or HTK	1 g/L	N	Controls (sham-operated); UW (CS 24 h); UW + 5 g/LM101 (CS 24 h); HTK (CS 24 h); HTK + 5 g/L M101 (CS 24 h)	Large white male pig	HBOCs lowered the peak of serum creatinine, reduced kidney inflammation levels, and maintained structural integrity; improved survival and function; and slowed the advance of interstitial fibrosis	Improved organ oxygenation and provided SOD activity	N
Mallet et al. (2014) Artif Organs 2014	M101	UW	1 g/L; 2 g/L; 5 g/L	N	UW solution (CS 24 h); UW+ 1 g/L M101 (CS 24 h); UW+ 2 g/L M101 (CS 24 h); UW+ 5 g/L M101 (CS 24 h)	Large white male pigs	Cells preserved better with HEMO2Life in a dose-dependent manner; better survival, metabolic activity, and cellular integrity *in vitro*; creatinine and fibrosis levels similar to those in pretransplant kidneys	Improved organ oxygenation	N
Mahboub et al. (2020) ARTIF ORGANS 2019	HBOC-201	UW	25%	Equilibrated with 95% O_2_ and 5% CO_2_, SaO_2_ > 97%	UW (37°C 30 min, CS 4°C 120 min, rewarming 90 min 10–37°C, anastomosis 30 min 21°C, reperfusion 37°C 120 min with HBOCs + UW); HBOCs (37°C 30 min, CS 4°C 120 min, rewarming 90 min 10–37°C with HBOCs, anastomosis 30 min 21°C, reperfusion 37°C 120 min with HBOCs)	Lewis rats	HBOCs improved kidney function and ultrafiltrate production, and improved glomerular filtration rate and sodium reabsorption	Improved organ oxygenation	Lack of transplantation validation; did not specifically study the O_2_ carrying capacity; Methemoglobin increased slightly and more during reperfusion
Aburawi et al. (2019) AM J TRANSPLANT 2019	HBOC-201	Williams E Media	3.5 g/dl	Equilibrated with 95% O_2_ and 5% CO_2_	HBOCs (6 h NMP); PRBCs (6 h NMP)	Human	HBOC/NMP system was feasible and did not result in inferior outcomes compared to PRBCs	Improved organ oxygenation	Relatively small sample sizes; lack of transplantation or a simulated reperfusion component
Kasil et al. (2019) Int. J. Mol. Sci 2019	M101	PERF-GEN^®^ preservation solution	2 g/L	N	Group W (1 h WI, 23 h HMP); Group W-O_2_ (1 h WI, 23 h HMP oxygenated with 100% O_2_ at 1 L/min); Group W-M10 (1 h WI, 23 h HMP+2 g/L M101); Group W-O_2_ + M101 (1 h WI, 23 h HMP oxygenated with 100% O_2_ at 1 L/min+2 g/L M101)	White pigs	M101 associated with or without 100% O_2_ improved kidney recovery and late graft outcome	Provided intrinsic SOD activity and regulated pO_2_; maintained ROS/anti-ROS balance	N
Bhattacharjee et al. (2020) TRANSPLANTATION 2019	HBOC-201	HTK	HBOC-201 and PlasmaLyte solution (1:2)	Oxygenated to 40%	SCS (30 min WI, CS HTK 8 h) 22°C blood (30 min WI, CS blood 4 h, blood SNMP 4 h) 22°C HBOCs (30 min WI, CS blood 4 h, HBOCs SNMP 4 h)	Landrace pigs	HBOC-201 had similar renal blood flow and function compared with blood; reduced acute tubular necrosis (ATN) scores and degrees of TUNEL staining; and reduced urinary damage markers and IL-6	Improved organ oxygenation; negative proinflammatory effect	N
Kaminski et al. (2019) Transpl Int 2019	M101	UW (CS) KPS (MP)	1 g/L or 2 g/L	N	CS-0 (60 min WI, CS UW 23 h ± 30 min); CS-1 (60 min WI, CS UW+ 1 g/L M101 23 h ± 30 min); CS-2 (60 min WI, CS UW+ 2 g/L M101 23 h ± 30 min) MP-0 (60 min WI, HMP KPS 23 h ± 30 min), MP-1 (60 min WI, HMP KPS+1 g/L M101 23 h ± 30 min); MP-2 (60 min WI, HMP KPS+2 g/L M101 23 h ± 30 min); SHAM (sham-operated); NEP (nephrectomized)	Large white male pigs	Cs arm: M101 dose-dependently improved long-term function MP arm: M101 improved short and long-term functional outcomes as well as tissue integrity	Improved organ oxygenation and increased ATP resynthesis and VEGF expression	N
Le Meur et al. (2020) Am J Transplant. 2020	M101	UW	1 g/L	N	M101 (CS or MP); UW (CS or MP). M101 added to the preservation solution of one of two kidneys from the same donor	Human	M101 significantly reduced DGF and improved renal function	Improved organ oxygenation	N
Pancreas									
Avila et al. (2006) Am J Transplant 2006	Polymerized human hemoglobin	Poly SFH-P	10 g/dl	Equilibrated with 100% O_2_ for 15 min	Poly SFH-P (30 min WI; 37°C 18 min perfusion); Control (30 min WI; 37°C 18 min perfusion)	Lewis rats	Poly SFH-P improved islet isolation oxygenation and preserved mitochondrial integrity	Improved organ oxygenation; improved islet viability; and improved integrity of both beta and non-beta cells	N
Espes et al. (2015) Transplantation 2015	PolyHb	Phosphate buffer (PB)	108.79 mg/ml	N	PolyHb (0.03 mg/g); PolyHb (0.1 mg/g); PolyHb (0.3 mg/g); Recipient: C57BL/6 nu/nu mice	Male C57BL/6	PolyHb effectively bridged the critical hypoxic phase immediately after transplantation, improved islet graft function, and reduced the number of islets needed for successful intramuscular transplantation	Improved organ oxygenation	Macrophages were attracted by the presence of the hemoglobin–haptoglobin complex in the high-dose treatment
Lemaire et al. (2019) J Cell Mol Med 2019	M101	2 g/L	Hanks’ Balanced Salt Solution (HBSS)	N	Cold storage (HBSS + M101 0, 2, 4, 6, 8, 12, and 18 h); Perfusion: 4°C 6 h with M101 or without M101	Wistar rat	M101 decreased oxidative stress (ROS) and necrosis (HMGB1); cellular stress pathway (p38 MAPK) activity was observed; improved post-isolation islet quality	Maintained oxidative phosphorylation; improved organ oxygenation; intrinsic SOD-like activity	N
Lemaire et al. (2019) J Cell Mol Med 2019	M101	2 g/L	UW	N	Cold storage (SCS 6 h + M101 3 h)	Human	M101 increased complex 1 mitochondrial activity and activated AKT activity; upregulated insulin secretion	Maintained oxidative phosphorylation; improved organ oxygenation; intrinsic SOD-like activity	N
Intestine									
Huang et al. (2014) Artif Cells Nanomed Biotechnol 2014	pPolyHb	UW	10.5 ± 0.5 g/dl	Equilibrated with 95% O_2_ and 5% CO_2_ for 30 min	Group 1: UW solution (SCS 12, 24, and 36 h); Group 2: HCA solution with 2 g/dl pPolyHb (SCS 12, 24, and 36 h); Group 3: HCA solution with 4 g/dl pPolyHb (SCS 12, 24, and 36 h); Group 4: HCA solution (SCS 12, 24, and 36 h)	Sprague–Dawley rats	Long-term (36 h) morphological integrity of the intestinal mucosa was better preserved in the pPolyHb; maintained tissue aerobic respiration and inhibited tissue anaerobic metabolism	Improved organ oxygenation	N

STS, St. Thomas’ solution; HS, hypothermic storage; CS, cold storage; NMP, normothermic machine perfusion; NO, nitric oxide; KHB, Krebs–Henseleit buffer; WI, warm ischemia; SOD, superoxide dismutase; HTK, histidine–tryptophan–ketoglutarate; KH-Sol, Krebs–Henseleit solution; ROS, reactive oxygen species; TLR, toll-like receptor; RBC, red blood cell; PRBC, packed red blood cells; CVR, cerebrovascular resistance; LPD, low-potassium dextran solution; EVLP, *ex vivo* lung perfusion; SnPP, Sn-protoporphyrin; HO-1, heme oxygenase-1; IPRL, isolated perfused rat liver; UW, University of Wisconsin; MP, machine perfusion; FFP, fresh frozen plasma; DHOPE, hypothermic oxygenated machine perfusion; COR, controlled oxygenated rewarming; SCS, static cold storage; ATP, adenosine triphosphate; SNMP, subnormothermic machine perfusion; HMP, hypothermic mechanical perfusion; KPS, kidney preservation solution; SFH-P, stroma-free hemoglobin pyridoxalated; HBSS, Hanks’ Balanced Salt Solution; HMGB1, high mobility group box; AKT, serine threonrine kinase; HCA, hypertonic citrate adenine.

### Heart

Compared to other solid organs, the clinically isolated heart has a relatively short safe cold storage time (up to 4–6 h). The heart is an aerobic organ that relies on a continuous supply of oxygen to produce high-energy phosphate ATP for mechanical functions. The supply of oxygen via HBOCs supports the resynthesis of ATP in mitochondria of isolated heart tissue, which delays the damage of ischemia and hypoxia and extends the cold storage time (Teh et al., 2017). Li et al. were the first to find that PolyPHb protected isolated hearts from IRI, which was related to a reduction in NO-mediated myocardial apoptosis and restoration of the nitroso-redox balance (Li et al., 2009). PolyPHb also improved mitochondrial function and maintained the mitochondrial structure, which indicated that it played an important role in maintaining redox homeostasis and reducing mitochondrial oxidative damage (Li et al., 2010). The pathogenesis of IRI is complex, and inhibition of the inflammatory response is a reasonable way to ameliorate IRI. PolyPHb protected isolated hearts from cold IRI by attenuating the expression of toll-like receptor 2 (TLR2) and toll-like receptor 4 (TLR4)/nuclear factor kappa B (NF-kB) signaling pathways (Wei et al., 2011). The addition of M101 to Celsior preservation solution significantly improved cardiac function recovery after ischemia, and the coronary blood flow and left ventricular development pressure (LVDP) recovered significantly after 60 min of reperfusion (Teh et al., 2017).

The studies mentioned above confirmed that HBOCs reduced the IRI of the isolated heart and improved aerobic metabolism and myocardial function. However, an HBOC-201–based perfusate restored myocardial energy storage and extracted oxygen faster than blood-based perfusion during *ex vivo* heart perfusion (EVHP) to meet the needs of myocardial metabolism, but it did not minimize myocardial injury or provide superior preservation of myocardial function (White et al., 2015). These effects may be due to HBOC-201–mediated reactive oxygen species (ROS) generation and the increase in methemoglobin (White et al., 2015), which caused cardiac endothelial cell injury and increased microvascular permeability and coronary vascular resistance (CVR) and ultimately led to myocardial edema and cardiac diastolic dysfunction. HBOC-201 may also have a unique mechanism of myocardial injury that leads to increased levels of troponin I and histological myocardial injury scores (White et al., 2015). Whether reducing the dose of HBOC-201 and incorporating additional antioxidants will optimize the preservation of the donor’s heart during EVHP is unclear and needs further verification in larger studies.

### Lung

Lung transplantation has been the most effective treatment for most end-stage lung diseases since 1983 (Reitz et al., 1983). IRI is the main reason for primary graft dysfunction (PGD), which is the major cause of mortality and morbidity in the postoperative period. During SCS of the isolated lung, high oxygen content in the alveoli increases the lipid peroxidation rate, and low oxygen content may lead to tissue hypoxia (Fukuse et al., 2001). In addition, the ideal condition for preserving the isolated lung is to provide enough oxygen to maintain the aerobic metabolism of the cells while minimizing hyperoxia-related cellular oxidant injury. M101 does not require any allosteric effector to release oxygen molecules in an oxygen gradient passively, and it provides an appropriate amount of oxygen to the tissues. Glorion et al. first used M101 as an additive to the standard preservation solution to prevent IRI during 24 h static cold preservation in a well-recognized single-lung allotransplantation model in pigs (Glorion et al., 2018). The functional parameters in the bronchoalveolar lavage fluid (BALF) and the arterial partial pressure of oxygen to fraction of inspired oxygen ratio (PaO_2_/FiO_2_) of the graft in the M101 group were significantly improved, and the graft vascular resistance was reduced after 5 h of reperfusion in the recipient. The study identified a circulating biomarker of ischemia–reperfusion, high mobility group box 1 protein (HMGB1). The expression of HMGB1 in the serum of the M101 group tended to be lower, and the activation of the HMGB1/RAGE (advanced glycation end products) axis in the control group explained the inflammatory response that led to PDG. The early serious events that occurred in the first few hours after transplantation were sufficient to capture the development of PDG. Therefore, the protective effect of M101 on IRI prevented the occurrence of PGD. Another study reported that SCS for up to 36 h after adding M101 reduced the risk of PGD after lung transplantation, which may be related to a significant decrease in circulating interleukin-6 (IL-6) levels (Ali et al., 2020). Compared to the standard lung preservation program, the isolated lung was washed and preserved with an M101 preservation solution and evaluated using *ex vivo* lung perfusion (EVLP) for 12 h; the M101 lung showed better lung function after transplantation. M101 has inherent oxidative stress reduction activity, but it produces no significant difference in oxidative injury. The effect of M101 in prolonging the isolated lung’s preservation time may not be as an antioxidant, but it may optimize tissue oxygenation in the area of atelectasis. Briefly, adding M101 preservation solution prolongs the preservation time of isolated lungs up to 48 h, which will help overcome long-distance obstacles and expand the donor pool.

### Liver

Liver graft quality depends on the donation status of the donor and the preservation effect during transportation from the donor to the recipient. During the preservation of the isolated liver, insufficient oxygen supply followed by reperfusion leads to an inflammatory network, and oxidative stress in the tissue leads to IRI. HBOCs induce the production of heme oxygenase 1 (HO-1) (Damle et al., 2009). Topp and Donner et al. confirmed that the positive effect of reducing isolated liver IRI depended on the induction of HO-1, which may be inhibited by tin protoporphyrin IX dichloride (SnPP) (Donner et al., 2013; Topp, et al., 2008). By inducing HO-1, HbG200 largely restored multidrug resistance–associated protein 2 (Mrp2) and bile salt export pump (Bsep) in pericentral hepatocytes and improved bile flow and biliary taurocholate excretion, which are related to diminished hepatocellular c-Jun N-terminal kinase (JNK) and Fyn signaling. Biliverdin/bilirubin and CO, which are the breakdown products of HO-1, also regulate JNK activity. Compared to traditional static cold preservation, both hypothermic oxygenated machine perfusion (HOPE) and SCS + M101 effectively oxygenated and increased ATP synthesis during the preservation of the isolated liver and prevented injury after liver transplantation (Alix et al., 2020). This study showed that HOPE had better results than M101 supplements, which may be related to the constant supply of oxygen and continuous capillary network flushing during liver perfusion. Nevertheless, SCS + M101 preservation for non–high-risk donor livers will be more convenient and easier to implement due to the high cost, manpower support, and risks of vascular injury associated with the use of HOPE. Compared to the current standard-of-care (CSP) system, the SNMP/HBOC system also significantly improved preservation of the isolated liver and provided superior graft function and 100% survival after transplantation, which may be further extended to the recovery and improvement of grafts obtained by ECDs (Fontes et al., 2015). In a pig model of DCD, HbV significantly increased the oxygen consumption of the donor’s liver during SNMP, but it did not prevent liver injury because the amount of HbV (0.6–0.7 g/dl) was insufficient (Shonaka et al., 2018). However, another study showed that SNMP + HbV solution reduced reperfusion injury in DCD donor livers (Shonaka et al., 2019). The mitochondria, pH, and lactate levels of the SNMP + HbV group were well maintained compared to the cold storage (CS), HMP, and SNMP groups.

NMP is generally performed using perfusion solutions based on RBCs to maintain physiological osmolarity, oncotic pressure, and adequate metabolic support. Laing et al. presented the first experience using an HBOC-based perfusion fluid to perfuse five discarded high-risk human livers in a human model of NMP (Laing et al., 2017). Livers perfused using the NMP/HBOC system had similar vascular flow parameters and perfused lactate clearance as the RBC-based perfusate but extracted more oxygen, which may be more beneficial in logistics, rheology, and immunology (Laing et al., 2017). Therefore, HBOC-201 may be used as an alternative oxygen carrier for RBCs in NMP. Matton et al. also reported that HBOC-201–based perfusate could be effective for NMP by using HBOC-201 + gelofusine perfusate instead of RBCs + FFP perfusate, while the results of some biomarkers of liver function and injury were even better without the use of human blood products (Matton et al., 2018). COR mitigates reperfusion injury and improves graft function posttransplantation (Hoyer et al., 2016). Boteon et al. reported that after perfusion of human high-risk donor livers using a combined cold-to-warm uninterrupted combined protocol (a single HBOC-based perfusate) and the HOPE + NMP interrupted combined protocol, both protocols reduced oxidation-mediated tissue injury and enhanced hepatic energy stores (Boteon et al., 2019). It showed that the combined protocol of COR and HBOC is beneficial for repairing high-risk donor livers while also eliminating the additional ischemic time required for perfusate exchange and simplifying the logistics of this combination, which may benefit its clinical application. de Vries et al. first reported that after serial DHOPE-COR-NMP using HBOC-201-based perfusate in seven high-risk donor livers, five of them were transplanted into recipients after evaluation that met all of the following transplantation criteria: within 150 min of NMP perfusate lactate <1.7 mmol/L, pH 7.35–7.45, bile production >10 ml, and bile pH > 7.45 (de Vries et al., 2019). Similarly, in a prospective clinical trial by van et al., after perfusion of 16 high-risk donor livers with HBOC-201-based perfusion solution combined with DHOPE-COR-NMP, 11 of these donor’s livers were transplanted into recipients after repair and evaluation of the liver and the survival of patients and grafts was 100% at 6 months (van Leeuwen et al., 2019). The study ultimately showed that the introduction of HBOCs in combination with DHOPE-COR-NMP could increase the number of high-risk donor liver transplants by 20% at the center. HBOC in combination with MP has been applied to the preservation of high-risk donor livers in clinical settings and has achieved a relatively favorable prognosis. However, randomized clinical trials with larger samples should be implemented to verify the benefits and prospects they offer, and the longer-term prognosis of the recipients should continue to be closely observed.

### Kidney

Kidney transplantation is the therapy of choice for end-stage renal diseases. IRI is a complex process that involves oxidative stress, mitochondrial uncoupling, and the coagulation cascade, which affect early graft function after organ transplantation and have an adverse effect on the long-term survival of grafts (Favreau et al., 2010). Thuillier et al. found that supplementation of M101 in UW or HTK solution reduced apoptosis and maintained mitochondria in a time- and dose-dependent manner during cold preservation in a pig kidney auto-transplantation model, with significant benefits in both early functional recovery and prognosis after transplantation, and reduced the occurrence of IRI and interstitial fibrosis and tubular atrophy (IFTA) (Thuillier et al., 2011). Similarly, Mallet et al. also presented a dose-dependent reduction in the extent of IRI and improved prognosis of grafts after cold preservation of porcine kidneys with UW solution supplemented with M101, and benefits were also obtained with lower doses of M101 (1 g/L) (Mallet et al., 2014). As high-risk donor kidneys are more prone to severe ischemia–reperfusion injury, there is an increased probability of delayed injury and dysfunction in the recipient and the graft (Oberbauer, 2016). As the demand for organ transplantation increases, repairing and assessing the quality of high-risk donor organs and improving the utilization of high-risk organs are among current solutions to expand the number of transplantable organs. Preservation of high-risk donor livers using SCS + M101 also showed dose-dependent improvement in long-term graft function; similarly, combining M101 with HMP had an improved effect on both short- and long-term graft function after transplantation (Kaminski et al., 2019). The combination of MP with HBOC allows for a uniform distribution of the HBOC in the vascular lumen and a more efficient delivery of oxygen to the tissues, further reducing ischemic and hypoxic injury and repairing the graft mass. HBOCs improve tissue oxygenation and repair kidney quality by releasing oxygen. However, if pure oxygen is added to the preservation solution for organ preservation, will the expected benefits be consistent? Kasil et al. evaluated the effect of supplemental oxygen [(100% O_2_) ± M101 (respectively or combined)] with HMP on the perfusion of isolated kidneys in a pig kidney auto-transplantation model (Kasil et al., 2019). The results showed that using 100% O_2_ in HMP without supplementation with M101 was harmful to the kidneys, which manifested as delayed vimentin expression and did not limit renal fibrosis. In addition, the combination of M101 and 100% O_2_ reduced the level of neutrophil gelatinase-associated lipocalin (NGAL) and maintained a high level of kidney injury molecule-1 (KIM-1) in the circulation. The study showed that supplementation with M101 with or without 100% O_2_ improves the HMP effect on kidney recovery and late graft results.

Gradual rewarming is a new perfusion modality in which HBOC maintains the preservation of perfusion of organs from hypothermia to normothermia. A transient increase in vascular resistance and methemoglobin was observed after preservation of the kidney using a preservation solution supplemented with HBOC-201 in a model of gradual rewarming but improved ultrafiltrate production, glomerular filtration rate, and sodium reabsorption during the reperfusion phase (Mahboub et al., 2020). In the context of combination with MP, the risk of HBOC-201 causing a transient increase in vascular resistance and methemoglobin transfer to the organ recipient is negligible because HBOC and methemoglobin will be washed out before transplantation. Perfusion preservation of organs using blood cannot be performed at low temperatures because it can lead to hemolysis and impair the quality of the grafts. Bhattacharjee et al. perfused porcine DCD kidneys with HBOC-201 or blood in combination with SNMP and found that tissue oxygen saturation, renal blood flow, and function were similar in both groups (Bhattacharjee et al., 2020). Thus, this demonstrated that HBOC-201 is a suitable oxygen carrier capable of replacing blood. NMP is capable of perfusing and repairing organs in combination with oxygen carriers and assessing the quality of the grafts. Mohamed et al. used HBOCs or packed red blood cells (PRBC) combined with NMP for perfusion and evaluation of human high-risk donor kidneys and found that the function and histological characteristics of the kidney were similar (Aburawi et al., 2019). This study confirmed that the HBOC solution could provide a logistically convenient alternative to PRBC in the NMP of human kidneys. Meur et al. demonstrated for the first time the safety and performance of the addition of M101 to the preservation solution of one of the two human kidneys from the same donor in a multicenter open-label study (Le Meur et al., 2020). This study showed that kidneys preserved with the addition of M101 showed no alterations in thrombosis or microcirculation on biopsy before implantation and 3 months after transplantation and had significantly less DGF and better kidney function after transplantation. The use of M101 for kidney preservation has been applied clinically and has confirmed the safety and promising efficacy data of M101 (Le Meur et al., 2020).

### Pancreas and Small Bowel

Pancreas or islet transplantation is a treatment for type 1 diabetes and pancreatitis (Barrou et al., 1994). During the preservation and isolation of islets, maintenance of proper oxygen levels is of great importance to prevent ischemia and reperfusion injury (Carlsson and Palm, 2002). Avila et al. showed that intratubular perfusion of HBOCs in the pancreas of rats undergoing 30 min of warm ischemia improved islet isolation and transplantation outcomes via maintaining mitochondrial integrity and did not lead to increased oxidative stress (Avila et al., 2006). M101 was added to the preservation solution during cold preservation of rat and human pancreas, and hypoxia and the oxidative stress of SOD were reduced, which alleviated tissue inflammation and necrosis (Lemaire et al., 2019). Overall, these results suggested that M101 had a positive effect on preserving the pancreas in rats and humans.

Muscle is a promising alternative site for islet transplantation, facilitating the rapid restoration of islet vasculature; however, it is susceptible to hypoxic limitation in the early posttransplant period. By co-transplanting islets with an oxygen carrier-PolyHb in mice, Daniel et al. found a dose-dependent reduction in β-cell hypoxia (Espes et al., 2015). Yet, the morphology of islets was significantly disrupted by the higher concentration of a bovine polyhemoglobin (PolyHb) inducing the aggregation of a large number of macrophages, and the reduction in β-cell apoptosis occurred only in the low-dose group (0.03 mg/g body weight). Lower doses of PolyHb co-transplanted with islets effectively bridged the critical hypoxic phase after transplantation, improved islet graft function, and reduced the number of islets required for successful intramuscular transplantation. In contrast, the inflammatory effect of higher concentrations of PolyHb may be due to the attraction of macrophages to the Hb–haptoglobin complex. HBOCs have also been used in rat small bowel preservation (Huang et al., 2014). This study suggested that hypertonic adenine citrate solution (HCA) combined with pPolyHb maintained tissue aerobic respiration and inhibited anaerobic metabolism with a preservation effect comparable to that of UW solution.

## Overall Conclusion and Future Perspectives

Due to the global shortage of organ resources, the imbalance of donor–recipient needs, and the increasing number of high-risk donors, these issues have led to considering what should be done to preserve grafts better. Although standard preservation solutions are currently used for static cold preservation, the repair and evaluation of marginal organs may rely on multiple combinations of MP, oxygenation strategies, and temperature regulation. Many efforts have been made by clinical practitioners and researchers to explore ways to mitigate graft ischemia–reperfusion injury, improve graft quality, and increase recipient and graft survival rates. Originally developed and utilized as a blood substitute, HBOCs are an ideal class of oxygen carriers with the ability to bind oxygen and release oxygen. HBOCs have now been extended to apply to tissues or organs in hypoxic conditions, where they act as an oxygen bridge to improve tissue oxygenation. The addition of HBOC to the preservation solution to improve the quality of the graft is a new promising strategy because they offer several advantages compared with traditional preservation solutions. First, HBOCs improve the oxygenation of isolated organs during SCS and enhance the quality of grafts. Second, the combination of HBOCs and MP provides a more targeted method, which can replace RBC perfusion to improve and evaluate the quality of high-risk grafts. In addition, preservation solutions containing HBOCs may be easily used in all solid organ transplants without further improving the existing preservation solution or perfusion platform protocols. Recent clinical trials have demonstrated the safety and efficacy of HBOCs in combination with mechanical perfusion platforms for liver and kidney preservation and improved prognosis for recipients and grafts.

Future prospects address the need for translational medicine research to determine the exact therapeutic dose and organ preservation time for human solid organs. Coordinated multicenter clinical trials are also needed in the subsequent clinical studies to accumulate larger, homogeneous cohorts. Additionally, some clinical trials with a randomized controlled design would be performed. This review summarizes the latest and most important research on HBOCs in solid organ preservation. Therefore, based on scientific evidence, HBOCs, one of the oxygen carriers for organ preservation, are ideal candidates for organ preservation additives. The mechanism of organ preservation by HBOCs has not been fully understood; the relevant mechanism would be investigated in depth in future studies to determine a more optimal preservation method. In addition, the vascular resistance, methemoglobin content, and oxidative damage index of grafts and recipients would also be closely monitored when using HBOCs. The future development of HBOCs may give a fascinating insight into the understanding of the safety and effectiveness of new strategies for solid organ preservation.

## Glossary

SOT: solid organ transplantation
DCD: donation after cardiac death
IRI: ischemia–reperfusion injury
ECD: expanded criteria donor
SCS: static cold storage
ATP: adenosine triphosphate
RBCs: red blood cells
RBC: red blood cell
PFCs: perfluorochemicals
SNMP: subnormothermic machine perfusion
NMP: normothermic machine perfusion
HBOCs: hemoglobin-based oxygen carriers
Hb: hemoglobin
Mb: myoglobin
MI: myocardial infarction
FDA: Food and Drug Administration
EA: expanded access
ECMO: extracorporeal membrane oxygenation
HbV: hemoglobin-vesicles
PolyPHb: polymerized human placenta hemoglobin
HbG: hemoglobin glutamer
PLP: pyridoxal phosphate
GDA: glutaraldehyde
PEG: polyethylene glycol
NO: nitric oxide
ET: endothelin
CO: carbon monoxide
EC: Euro-Collins
UW: University of Wisconsin
HTK: histidine–tryptophan–ketoglutarate
DHOPE: dual hypothermic oxygenated machine perfusion
COR: controlled oxygenated rewarming
MP: machine perfusion
TLR2: toll-like receptor 2
TLR4: toll-like receptor 4
NF-kB: nuclear factor kappa B
LVDP: left ventricular development pressure
EVHP: *ex vivo* heart perfusion
ROS: reactive oxygen species
CVR: coronary vascular resistance
GMP: good manufacturing practice
SOD: superoxide dismutase
MDA: malondialdehyde
PGD: primary graft dysfunction
BALF: bronchoalveolar lavage fluid
PaO_2_/FiO_2_: arterial partial pressure of oxygen to fraction of inspired oxygen ratio
HMGB1: high mobility group box 1
RAGE: advanced glycation end products
EVLP: *ex vivo* lung perfusion
HO-1: heme oxygenase 1
SnPP: Tin protoporphyrin IX dichloride
CS: cold storage
Mrp2: multidrug resistance–associated protein 2
Bsep: bile salt export pump
JNK: c-Jun N-terminal kinase
HOPE: hypothermic oxygenated machine perfusion
CSP: current standard-of-care
HMP: hypothermic machine perfusion
IFTA: interstitial fibrosis and tubular atrophy
NGLA: neutrophil gelatinase–associated lipocalin
KIM-1: kidney injury molecule-1
PRBC: packed red blood cell
PolyHb: polyhemoglobin
HCA: hypertonic adenine citrate solution
